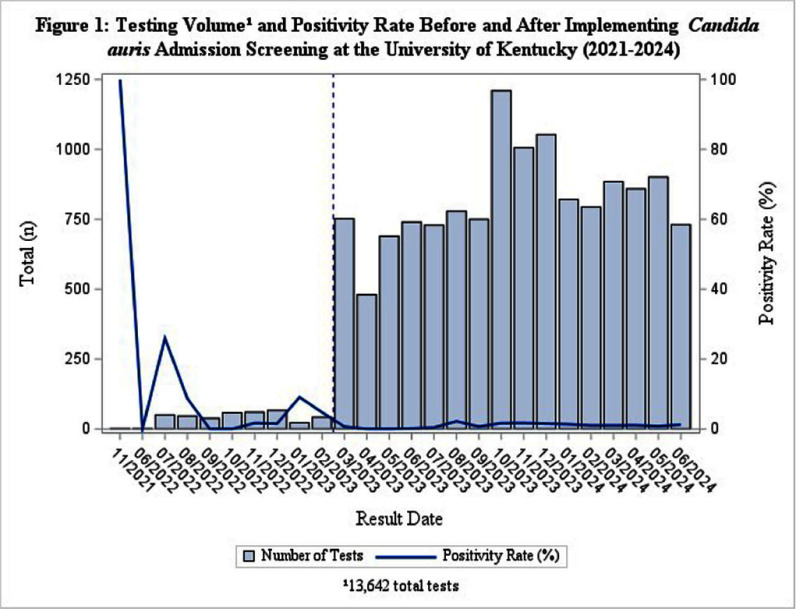# Candida auris Screening, Positivity Trends, and Patient Characteristics at the University of Kentucky between 2021 and 2024

**DOI:** 10.1017/ash.2025.329

**Published:** 2025-09-24

**Authors:** Faith Fursman, Natalie Fitzsimmons, Court Desmond, Kimberly Blanton, Kevin Hatton, Rachel Howard, DaNelle Overton, Olafsson David, Sean McTigue, Nicholas Van Sickels, Takaaki Kobayashi

**Affiliations:** 1 University of Kentucky Healthcare; 2 UK Healthcare; 3 University of Kentucky

## Abstract

**Background:** Candida auris is an emerging multidrug-resistant fungus recognized as a global health threat. Despite increasing rates of colonization, no standardized protocol exists in the United States for C. auris screening upon admission. In February 2023, the University of Kentucky Healthcare (UKHC) implemented a targeted C. auris screening system for select high-risk patients. **Methods:** This retrospective observational study was conducted at UKHC, a 1,086-bed academic medical center, using data from patients aged ≥18 years screened for C. auris between July 1, 2021, and June 30, 2024. Prior to February 2023, C. auris screening occurred only during outbreak investigations. Post-implementation, screening was expanded to include ICU admissions, patients from external facilities with wounds or tracheostomies, and patients with a history of carbapenem-resistant organism infection. Axillary and groin swabs were tested via polymerase chain reaction (PCR). Cases were classified as community-onset (CO) **Results:** Of 13,642 C. auris tests performed, 70 positive cases were identified: 13 cases (6 CO, 7 HO) pre-implementation and 57 cases (31 CO, 26 HO) post-implementation (Figure 1). The mean age was 60.24 years, and males comprised 57.75%. The monthly positivity rate post-implementation ranged from 0% to 2.18% (with a mean of 0.96%). Among the 70 cases, 10 (14.29%) were classified as clinical infections, and 60 (85.71%) as colonization. The primary indications for C. auris screening included ICU admission (42.86%), point prevalence surveys (17.14%), and admission from external facilities with wounds (5.72%). No significant differences were observed between clinical and colonized cases by age, gender, race, or most other comorbidities. However, clinical cases were more likely to have diabetes (90% vs. 48.33%, p=0.0143) and medical device usage, including tracheostomy (80% vs. 45.00%, p=0.0404), gastrostomy tubes (90% vs. 53.33%, p=0.0293), central lines (60% vs. 41.67%, p=0.2799), and urinary catheters (60% vs. 46.67%, p=0.4348). Among ten clinical cases, seven patients received antifungal treatment. Three patients did not receive any treatment since C. auris was not considered clinically significant. 30-day mortality was higher among clinical cases compared to colonized cases; however, the difference was not statistically significant (30% vs. 25%, p=0.7377). **Conclusions:** The implementation of a targeted C. auris screening program at UKHC has provided critical insights into epidemiologic trends, patient demographics, and risk factors. Understanding these factors is essential for optimizing infection prevention strategies, refining screening protocols, and informing public health efforts to mitigate the spread of C. auris in healthcare settings.

**Figure:**